# Supplementary material for: Association between Achievement of Estimated Average Glucose Level and 6-Month Neurologic Outcome in Comatose Cardiac Arrest Survivors: A Propensity Score-Matched Analysis
Source: J Clin Med. 2019 Sep 18;8(9):1480. doi: 10.3390/jcm8091480 (PMC6780944; doi:10.3390/jcm8091480)
Supplement: Supplementary file 1 [file jcm-08-01480-s001.pdf]

Supplementary

Table S1. Univariate and multivariate logistic regression analyses for unfavorable neurologic outcome in entire cohort.

	Crude OR (95% CI)	*p*	Adjusted OR (95% CI)	*p*
Age, years	1.046 (1.030–1.062)	<0.001	1.042 (1.019–1.065)*	<0.001
Male	0.559 (0.352–0.886)	0.013	0.579 (0.297–1.127)	0.108
Coronary artery disease	0.866 (0.499–1.501)	0.607	NA	
Congestive heart failure	1.173 (0.570–2.416)	0.665	NA	
Hypertension	1.556 (1.021–2.403)	0.040	0.739 (0.363–1.505)	0.404
Diabetes	3.157 (1.870–5.329)	<0.001	1.054 (0.443–2.511)	0.905
Pulmonary disease	2.888 (0.821–10.158)	0.098	1.843 (0.323–10.515)	0.491
Renal impairment	3.116 (1.414–6.868)	0.005	1.346 (0.460–3.941)	0.587
Cerebrovascular accident	1.636 (0.674–3.974)	0.277	NA	
Hepatic disease	2.810 (0.325–24.299)	0.348	NA	
Body mass index, kg/m^2	0.976 (0.925–1.030)	0.383	NA	
OHCA	0.908 (0.517–1.593)	0.735	NA	
Witnessed	0.342 (0.197–0.596)	<0.001	0.593 (0.268–1.312)	0.197
Bystander CPR	0.628 (0.404–0.979)	0.040	1.049 (0.523–2.104)	0.892
Shockable rhythm	0.107 (0.066–0.174)	<0.001	0.246 (0.114–0.531)*	<0.001
Cardiac etiology	0.179 (0.109–0.292)	<0.001	0.268 (0.126–0.567)*	0.001
Adrenaline, mg	1.207 (1.107–1.316)	<0.001	0.931 (0.831–1.042)	0.211
Time to ROSC, min	1.042 (1.026–1.058)	<0.001	1.062 (1.038–1.087)*	<0.001
HgA1c, %	1.544 (1.224–1.948)	<0.001	1.411 (1.049–1.897)*	0.023
Hemoglobin, mg/dL	0.762 (0.694–0.837)	<0.001	0.923 (0.796–1.070)	0.289
Lactate, mmol/L	1.124 (1.062–1.189)	<0.001	1.100 (1.012–1.195)*	0.025
Glucose, mg/dL	1.003 (1.001–1.005)	0.004	1.001 (0.997–1.005)	0.598
PaO_2, mmHg	1.003 (1.001–1.005)	0.013	1.005 (1.001–1.008)*	0.010
PaCO2, mmHg	1.013 (0.997–1.029)	0.109	1.018 (0.993–1.043)	0.155
Glasgow Coma Scale	0.587 (0.493–0.698)	<0.001	0.737 (0.595–0.913)*	0.005
SOFA score	1.215 (1.134–1.302)	<0.001	1.091 (0.986–1.206)	0.090
Time from ROSC to TH, min	1.002 (1.000–1.004)	0.046	1.003 (1.000–1.006)*	0.029
Induction duration, h	0.737 (0.656–0.830)	<0.001	0.843 (0.716–0.994)*	0.042
Rewarming duration, h	1.107 (1.026–1.195)	0.009	1.028 (0.920–1.148)	0.631

OR, odds ratio; CI, confidence interval; NA, not applicable; OHCA, out-of-hospital cardiac arrest; CPR, cardiopulmonary resuscitation; ROSC, restoration of spontaneous circulation; HbA1c, glycated hemoglobin; PaO2, partial pressure of oxygen; PaCO2, partial pressure of carbon dioxide; SOFA, sequential organ failure assessment; TH therapeutic hypothermia.* Variables included in the final model.

Table S2. Univariate and multivariate logistic regression analyses for unfavorable neurologic outcome in matched cohort.

	Crude OR (95% CI)	*p*	Adjusted OR (95% CI)	*p*
Age, years	1.029 (1.007–1.051)	0.010	1.017 (0.985–1.050)	0.295
Male	0.563 (0.274–1.157)	0.118	0.870 (0.297–2.547)	0.800
Coronary artery disease	0.812 (0.343–1.923)	0.635	NA	
Congestive heart failure	3.062 (0.675–13.896)	0.147	4.633 (0.741–28.968)	0.101
Hypertension	0.760 (0.398–1.451)	0.405	NA	
Diabetes	1.852 (0.848–4.045)	0.122	0.717 (0.223–2.304)	0.779
Pulmonary disease	1.750 (0.365–8.389)	0.484	NA	

Renal impairment	1.851 (0.509–6.732)	0.350	NA	
Cerebrovascular accident	1.561 (0.422–5.776)	0.505	NA	
Hepatic disease	NA		NA	
Body mass index, kg/m^2	1.008 (0.932–1.092)	0.837	NA	
OHCA	1.188 (0.521–2.708)	0.682	NA	
Witnessed	0.154 90.052–0.454)	0.001	0.364 (0.093–1.435)	0.149
Bystander CPR	0.324 (0.150–0.700)	0.004	0.306 (0.102–0.912)*	0.034
Shockable rhythm	0.087 (0.041–0.184)	<0.001	0.056 (0.020–0.159)*	<0.001
Cardiac etiology	0.189 (0.089–0.400)	<0.001	0.560 (0.163–1.925)	0.358
Adrenaline, mg	1.174 (1.024–1.346)	0.021	0.843 (0.696–1.020)	0.079
Time to ROSC, min	1.037 (1.014–1.061)	0.001	1.080 (1.036–1.126)*	<0.001
HgA1c, %	1.385 (0.935–2.051)	0.104	1.264 (0.757–2.109)	0.370
Hemoglobin, mg/dL	0.803 (0.700–0.921)	0.002	0.803 (0.658–0.980)*	0.031
Lactate, mmol/L	1.077 (0.991–1.171)	0.080	1.066 (0.951–1.194)	0.270
Glucose, mg/dL	0.998 (0.995–1.002)	0.404	NA	
PaO_2, mmHg	1.004 (1.000–1.008)	0.029	1.007 (1.001–1.013)*	0.017
PaCO2, mmHg	1.013 (0.990–1.036)	0.278	NA	
Glasgow Coma Scale	0.618 (0.477–0.802)	<0.001	0.891 (0.617–1.287)	0.539
SOFA score	1.141 (1.030–1.264)	0.012	1.205 (1.016–1.430)*	0.033
Time from ROSC to TH, min	1.002 (0.999–1.004)	0.223	NA	
Induction duration, h	0.777 (0.639–0.945)	0.011	1.039 (0.738–1.464)	0.825
Rewarming duration, h	1.012 (0.890–1.1520	0.852	NA	

OR, odds ratio; CI, confidence interval; NA, not applicable; OHCA, out-of-hospital cardiac arrest; CPR, cardiopulmonary resuscitation; ROSC, restoration of spontaneous circulation; HbA1c, glycated hemoglobin; PaO2, partial pressure of oxygen; PaCO2, partial pressure of carbon dioxide; SOFA, sequential organ failure assessment; TH therapeutic hypothermia. * Variables included in final model.
